# Rapid Evolution of Pandemic Noroviruses of the GII.4 Lineage

**DOI:** 10.1371/journal.ppat.1000831

**Published:** 2010-03-26

**Authors:** Rowena A. Bull, John-Sebastian Eden, William D. Rawlinson, Peter A. White

**Affiliations:** 1 School of Biotechnology and Biomolecular Sciences, Faculty of Science, University of New South Wales, Sydney, New South Wales, Australia; 2 Virology Division, SEALS, Department of Microbiology, Prince of Wales Hospital, Randwick, Sydney, New South Wales, Australia; Centro de Biología Molecular Severo Ochoa (CSIC-UAM), Spain

## Abstract

Over the last fifteen years there have been five pandemics of norovirus (NoV) associated gastroenteritis, and the period of stasis between each pandemic has been progressively shortening. NoV is classified into five genogroups, which can be further classified into 25 or more different human NoV genotypes; however, only one, genogroup II genotype 4 (GII.4), is associated with pandemics. Hence, GII.4 viruses have both a higher frequency in the host population and greater epidemiological fitness. The aim of this study was to investigate if the accuracy and rate of replication are contributing to the increased epidemiological fitness of the GII.4 strains. The replication and mutation rates were determined using *in vitro* RNA dependent RNA polymerase (RdRp) assays, and rates of evolution were determined by bioinformatics. GII.4 strains were compared to the second most reported genotype, recombinant GII.b/GII.3, the rarely detected GII.3 and GII.7 and as a control, hepatitis C virus (HCV). The predominant GII.4 strains had a higher mutation rate and rate of evolution compared to the less frequently detected GII.b, GII.3 and GII.7 strains. Furthermore, the GII.4 lineage had on average a 1.7-fold higher rate of evolution within the capsid sequence and a greater number of non-synonymous changes compared to other NoVs, supporting the theory that it is undergoing antigenic drift at a faster rate. Interestingly, the non-synonymous mutations for all three NoV genotypes were localised to common structural residues in the capsid, indicating that these sites are likely to be under immune selection. This study supports the hypothesis that the ability of the virus to generate genetic diversity is vital for viral fitness.

## Author Summary

Since 1995, norovirus has caused five pandemics of acute gastroenteritis. These pandemics spread across the globe within a few months, causing great economic burden on society due to medical and social expenses. Norovirus, like influenza virus, has over 40 genotypes circulating within the population at the same time. However, it is only a single genotype, known as genogroup II genotype 4 (GII.4), that causes mass outbreaks and pandemics. Very little research has been conducted to determine why GII.4 viruses can cause pandemics. Consequently, we compared the evolution properties of several pandemic GII.4 strains to non-pandemic strains and found that the GII.4 viruses were undergoing evolution at a much higher rate than the non-pandemic norovirus strains. This phenomenon is similar to influenza virus, where an increase in antigenic drift has been associated with increased outbreaks. This discovery has important implications in understanding norovirus incidence and also the development of a vaccine and treatment for norovirus.

## Introduction

Norovirus (NoV), a member of the *Caliciviridae* family, is now considered the most common cause of viral gastroenteritis outbreaks in adults worldwide [1]. In the US, NoV has been identified as the cause of over 73% of outbreaks of gastroenteritis [1]. Furthermore, outbreak NoV strains spread rapidly causing great economic burden on society due to medical and social expenses. Consequently, a vaccine or treatment for NoV would be useful in reducing its transmission and alleviating disease symptoms. Our current knowledge of NoV replication and evolution has made it difficult to predict the efficacy of a treatment or longevity of a vaccine, as evidence is emerging that NoV, like many other RNA viruses, exists as a dynamic, rapidly evolving and genetically diverse population [2],[3],[4]. The high level of genetic diversity in RNA viruses is recognised as the basis for their ubiquity and adaptability [5]. Therefore, in order to develop a successful treatment or control program it is first necessary to understand the mechanisms behind NoV replication and evolution.

NoV is a small round virion of 27–38 nm in diameter and possesses a single-stranded, positive-sense, polyadenylated, RNA genome of 7400–7700 nucleotides [6]. The human NoV genome is divided into three open reading frames (ORFs). ORF1 encodes for the non-structural proteins, including an NTPase, 3C-like protease and RNA-dependent RNA polymerase (RdRp) [7]. The two structural proteins VP1, the major capsid protein, and VP2, the minor capsid protein are encoded by ORF2 and ORF3, respectively [8],[9].

NoV is a highly diverse genus with up to 61% VP1 amino acid diversity between its five genogroups (GI to GV) [10]. Up to 44% amino acid diversity over VP1 is also observed within the genogroups and has resulted in the further subgrouping of GI, GII and GIII into 8, 17 and 2 genotypes, respectively [10]. VP1 exhibits the highest degree of sequence variability in the genome [11],[12]. It consists of three domains, namely the shell (S) domain connected by a flexible hinge (P1 domain) to a protruding domain (P2) [13]. The highly conserved S domain forms the backbone of the capsid structure [13], while the moderately conserved P1 domain encodes the flexible hinge that connects the S and P2 domains. The protruding P2 domain possesses motifs that are involved in binding to the host cell, and hence, the P2 domain is responsible for the antigenicity of the virus [14],[15].

The most clinically significant of the five genogroups is GII, as it is the most prevalent human NoV genogroup detected and more frequently associated with epidemics compared with other genogroups. Of particular interest is GII genotype 4, (GII.4), because this lineage accounts for 62% of all NoV outbreaks globally [14],[15] and has also caused all five major NoV pandemics in the last decade (1995/1996, U5-95_US strain; 2002, Farmington Hills; 2004, Hunter; 2006, 2006a virus; and 2007, 2006b virus) [16],[17],[18],[19]. The basis for the increased epidemiological fitness [20] of the GII.4 strains, as determined by its high incidence and ability to cause pandemics, is currently unknown. Investigations with influenza indicate a link between increased viral evolution and increased viral incidence [21],[22]. However, because of the non-culturable nature of human NoV, variations in rates of evolution have not been calculated for different NoVs and consequently this has not been investigated as a factor in determining viral incidence and epidemiological fitness.

Replication efficiency and genetic diversity are both important parameters in viral fitness [23]. The aim of this study was to determine if these two parameters are contributing to the increased epidemiological fitness of the GII.4 strains. Replication efficiency and genetic diversity are primarily determined by the viral RdRp, as it controls the rate new sequence is introduced into the genome. Therefore using *in vitro* RdRp assays together with bioinformatics, the replication efficiency, mutation rate and rate of evolution of GII.4 viruses was compared with other NoV GII genotypes. The results of this study suggest that, like influenza A, the increased incidence of the pandemic GII.4 lineage may be a result of the combined influence of a high mutation, replication and evolution rate which, together culminate in an increased epidemiological fitness for the GII.4 strains.

## Materials and Methods

### NoV strains

Stool samples containing NoV were obtained from the Department of Microbiology, Prince of Wales Hospital, Sydney, Australia, with the exception of the stool specimen that contained NoV/Mc17/01/Th (GenBank accession numbers AY237413). This stool specimen was obtained from McCormic Hospital, Chiang Mai, Thailand [16].

The six genetically diverse NoV strains used in this study included: three GII.4 pandemic strains; NoV/Sydney 348/97/AU (of the NoV/US95_96 GII.4 pandemic lineage) [16], NoV/NZ327/06/NZ (NoV/2006a GII.4 lineage) [17] and NoV/NSW696T/06/AU (NoV/2006b GII.4 lineage) [17]. Two recombinant strains; NoV/Sydney C14/02/AU (GII.b ORF1 and GII.3 ORF2/3 [commonly referred to as GII.b/GII.3]) [16] and NoV/Sydney4264/01/AU (GII.4 ORF1 and GII.10 ORF2/3, [GII.4/GII.10]) [16], and a GII.7 NoV, NoV/Mc17/01/Th associated with rare sporadic cases of gastroenteritis [24]. In this study, the RdRp enzymes are referred to by their genotype, except in the case of the GII.4 strains, which are referred to by their pandemic name, eg. GII.4 2006b-RdRp (see Table 1). RdRps from recombinant strains are indicated by an ‘r’ in front of the nomenclature.

**Table 1:** Comparison of the replication accuracy and rate for NoV and HCV RdRps.

RdRp^a^	Derivative Strain	*K_cat_* (s^−1^)		Mutation Rate		Rate of Evolution		U^c^ (subs/genome)
				*in vitro* nt subs/site^d^		*sequence data* nt subs/site/yr		
		Mean^d^	SD	Mean^d^	SD	Mean	SD	
GII.4 2006b	NoV/Sydney696T/06/AU	0.209	0.054	9.06×10^−4^	2.88×10^−4^	3.9×10^−3^	2.4×10^−4^	6.80
rGII.4	NoV/Sydney4264/06/AU	0.168	0.024	8.34×10^−4^	2.56×10^−4^	Not done		6.26
GII.4 US95/96	NoV/Sydney348/97/AU	0.158	0.039	8.87×10^−4^	4.42×10^−4^	3.9×10^−3^	2.4×10^−4^	6.65
GII.4 2006a	NoV/NZ327/06/AU	0.183	0.024	5.54×10^−4^	3.84×10^−4^	3.9×10^−3^	2.4×10^−4^	4.16
rGII.b	NoV/SydneyC14/02/AU	0.155	0.093	1.53×10^−4^	1.22×10^−4^	2.4×10^−3^	4.9×10^−4^	1.15
GII.7	NoV/Mc17/02/Th	0.238	0.088	2.21×10^−5^	4.58×10^−4^	2.3×10^−3^	1.5×10^−4^	0.17
HCV/3a	HCV/VRL69	0.003	8.06×10^−5^	1.97×10^−3^	1.42×10^−3^	Not done		18.57
HCV/1b	HCV/VRL75	0.003	1.06×10^−4^	1.23×10^−3^	1.14×10^−3^	2.3×10^−2^ b	N/A	11.57

a The RdRps are named after their genotype except for the GII.4 pandemic strains which are named after their subcluster. Recombinants are indicated by an r before the genotype.

b HVR1, published by Rispeter et al. [53].

c U: Number of mutations per viral replication round.

d The mean and standard deviation of a triplicate data set.

### RNA extraction and cDNA synthesis

Viral RNA was extracted from 140 µl of 20% faecal suspension using the QIAmp Viral RNA kit according to manufacturers' instructions (Qiagen, Victoria, Australia). RNA was resuspended in 50 µl of Baxter Steri-pour H_2_O and stored at −80°C. cDNA synthesis was performed as described previously [16].

### Amplification of capsid and RdRp regions

The full length capsid gene, P2 domain and RdRp regions were amplified with specific primers (Table 2) using reverse transcriptase - polymerase chain reaction (RT-PCR) methods described in [17]. The amplified RdRp genes were cloned into pGEM-T Easy vector (Promega, Wisconsin, United States).

**Table 2:** Oligonucleotide sequences designed and used in this study.

Primer	Region	Genotype	Polarity^a^	Sequence 5′-3′ b
GV6	5′ Capsid	GII	−	TTRTTGACCTCTGGKACGAG
GV11	5′ RdRp	GII.4 US95_96	+	CTAGGATCCAGGTGATGACAGTAAGGGAAC
GV12	3′ RdRp	GII.4 US95_96	−	TCAGAATTCGAYTCGACGCCATCTTCATTCTCA
GV21	3′ Protease	GII	+	GTBGGNGGYCARATGGGNATG
GV23	5′ RdRp	rGII.b, GII.4 2006a	+	CGCGGATCCAGGTGGCGACAACAAGGGAA
GV24	3′ RdRp	rGII.b, GII.4 2006a, GII.4 2006b	−	CCGGAATTCGATTCGACGCCATCTTCATTCACA
GV37	5′ RdRp	GII.7	+	GACGAGCTCGGGAAATCAGGACCTT
GV38	3′ RdRp	GII.7	−	CCCAAGCTTGGATTCGACGCCATC
GV40	3′ RdRp	rGII.4	−	GCCTGCAGTACTTCGACGCCATC
GV43	P2	GII.4	+	YAGCCCYGAYTTYTCRTT
GV44	P2	GII.4	−	ARRTGYTGNAYCCAYTCYTG
GV171	3′ RdRp	GII.4 2006b	−	CGCGGATCCAGGTGGTGACAGTAAGGG
GV172	5′ RdRp	rGII.4	+	CGCGGATCCAGGCGGTGACAACAAAGG
GV194	K291T	GII.4 US95_96	+	GGTGACTTCACAATATCAATC
GV195	K291T	GII.4 US95_96	−	GATTGATATTGTGAAGTCACC
GV196	T291K	GII.4 2006a	+	GGTGACTTCAAAATATCAATC
GV197	T291K	GII.4 2006a	−	GATTGATATTTTGAAGTCACC

a “+” indicates that the oligonucleotide is a forward primer and “−” indicates that the oligonucleotide is a reverse primer.

b Underlined sections indicate restriction enzyme sites.

### DNA sequencing

Plasmids and PCR products were purified by PEG precipitation and washed with 70% ethanol. Products were sequenced directly on an ABI 3730 DNA Analyzer (Applied Biosystems, Foster City, CA, US) using dye-terminator chemistry.

### Construction of RdRp expression vectors and sequence mutagenesis

pGEM-T Easy vectors containing 1736 bp from the 3′ end of ORF1 were purified using the Quantum prep® plasmid miniprep kit (BioRad, California, United States) and used as template DNA for the construction of expression vectors. Strain specific primers incorporating restriction enzyme sites, were designed to amplify the precise RdRp region of each strain (Table 2). PCR was performed as described previously [17]. PCR products were digested with their corresponding restriction enzymes and cloned into the expression vector pTrcHis2A (Invitrogen, Mount Waverley, Australia). Constructs containing the hepatitis C virus (HCV) genotype 3a RdRp (pVRL69) and HCV genotype 1b RdRp (pVRL75), were used as controls and have been described previously [25].

Site directed mutagenesis of residue 291 in the GII.4 US95_96-RdRp and the GII.4 2006a-RdRp was carried out with the Stratagene Quickchange II mutagenesis kit, according to manufacturer's instructions (Stratagene, La Jolla, United States). The primers used to introduce the mutation into the plasmid are listed in Table 2.

### RdRp expression and purification

The NoV RdRps and control HCV RdRps were expressed in *Escherichia coli*, as described previously [25], except expression of the NoV RdRps was performed for 4 hr at 30°C. Purity was checked by SDS-PAGE and the identity of the RdRp was confirmed by western blot with an anti-six histidine antibody and peptide sequencing performed by the Bioanalytical Mass Spectrometry Facility (University of New South Wales, Australia). Recombinant RdRp was quantified with a Nanodrop ND-1000 Spectrophotometer (Nanodrop, Wilmington, United States).

### RdRp kinetic measurements

Kinetic RdRp assays were performed in a final volume of 15 µl and contained 20 mM Tris-HCl (pH 7.4), 2.5 mM MnCl_2_, 5 mM DTT, 1 mM EDTA, 500 ng of homopolymeric C RNA template, 2 U RNasin (Promega), 4 mM sodium glutamate and increasing concentrations of [^3^H]-GTP (Amersham Biosciences, Little Chalfont, UK) ranging from 2 µM to 60 µM. Reactions were initiated with the addition of 50 nM of RdRp and incubated for 9 mins at 25°C. The reactions were terminated by adding EDTA to a final concentration of 60 mM, 10 µg herring sperm DNA and 170 µl of 20% (w/v) trichloroacetic acid. The incorporated radionucleotides were precipitated on ice for 30 min and then filtered through a 96 well GF/C unifilter microplate (Falcon, Franklin Lakes, United States) by a Filtermate harvester (Packard BioSciences, Melbourne, Australia). Using the harvester, the filters were washed thoroughly with water and left to dry. The filter wells were each filled with 25 µl of Microscint scintillation fluid (Packard Biosciences) and radioactivity measured using a Packard liquid scintillation counter (TopCount NXT; Packard Biosciences). Background measurements for each assay consisted of reactions without RdRp and were subtracted from the count per minute (CPM) values obtained for the individual enzyme assays. Results were plotted and statistical analysis performed with the Mann Whitney Test (one-tailed, 95% confidence interval) in GraphPad Prism version 4.02 (GraphPad Software, San Diego, CA).

### Incorporation fidelity

An *in vitro* fidelity assay was developed to measure mutation rates and was adapted from Ward *et al.* [26]. The RdRp assay was performed using conditions described above with a homopolymeric C RNA template, except 82.1 pmoles of [^3^H]UTP (2 µCi) or [^3^H]ATP (4 µCi) (Amersham Biosciences) were added (as the non-complementary nucleotides) with an equimolar amount of GTP (82.1 pmoles) (Promega) added as the complementary nucleotide. The total amount of ribonucleotide incorporated was calculated in a parallel experiment with the addition of 1 µCi (164.2 pmoles) [^3^H]GTP (Amersham Biosciences) as the correct nucleotide. The assay was incubated for 50 min at 25°C. Error frequency of the RdRp was determined by calculating the total number (pmoles) of non-complementary ribonucleotides incorporated and dividing by the total number (pmoles) of [^3^H]GTP ribonucleotides incorporated.

### Evolutionary analysis of NoV capsids

In order to determine the rate of evolution of the rGII.3, GII.3, GII.4 and GII.7 capsids, the nucleotide sequences of ORF2 were analysed. RNA capsid sequences used for the analysis included eight from this study and 76 sequences from GenBank, with the oldest strains available dating back to 1987. The strains used and their GenBank accession numbers are listed in Text S1. The rate of evolution (substitutions/nucleotide site/year) for GII.3, GII.b/GII.3 GII.4 and GII.7 NoVs was determined by calculating the number of nucleotide substitutions in ORF2 compared to an ancestral strain and this was plotted against time [27]. The rate of evolution was determined by linear regression with the program GraphPad PRISM® version 4 and was equivalent to the gradient of the line. Pairwise alignments of RNA sequences and evolutionary distances between sequences were carried out using the Maximum Composite Likelihood model in Mega 4.0 [28]. Bootstrapped trees (1000 data sets) were constructed using the Neighbour-joining method, also with the program Mega 4.0.

In order to determine the amount of selection each genotype is under, the average Ka/Ks ratio was calculated for each genotype's capsid gene (GII.4, GII.b/GII.3 and GII.7). The Ka/Ks ratio is a measure of nonsynonymous amino acid changes compared to synonymous (silent) changes. Ka/Ks>1 indicates that positive selection is occurring. Ka/Ks = 1 is interpreted as neutral evolution and Ka/Ks<1 is indicative of negative or purifying selection. The program Sliding Windows Alignment Analysis Program (SWAAP) version 1.0.2 [29] was utilised. The Nei-Gojobori model was used to calculate Ka and Ks values [30]. The window size was set at 15 bp (5 aa) and the step size was 3 bp (1 aa).

### Protein modelling

Predicted secondary structure analysis of the RdRps and capsid protein VP1 were performed by generating a Protein Data Bank (PDB) file from the amino acid sequence in FastA format using software on the CPHmodels 2.0 Server [31]. Three dimensional structures were then generated from the PDB files with PyMol [32].

### Accession numbers

The GenBank accession numbers for the RdRp and capsid genes described in this paper are listed in Text S1.

## Results

### In vitro analysis of NoV RdRps

#### NoV selection, expression and purification of RdRps

To investigate the replication efficiency and mutation rate for different NoV and control HCV RdRps, the RdRp encoding region from six NoVs and two HCVs were cloned and expressed. NoVs selected for this study included three pandemic GII.4 strains; a non-pandemic recombinant virus with a GII.4 ORF1 (RdRp) and a GII.10 ORF2/3 (capsid) (GII.4/GII.10), a second recombinant virus GII.b/GII.3, the second most prevalent strain [16], and a rarely detected GII.7 strain. The RdRps were expressed with additional N-terminal amino acids MDP and a C-terminal *myc* epitope and hexahistidine tag. Approximately 1 to 3 mg of a 60 kDa and 68 kDa enzyme, for NoV and HCV, respectively, were obtained and confirmed by mass spectrometry (data not shown).

#### Replication rate

To determine the nucleotide incorporation rate for the six NoV RdRps and the two HCV RdRps, the *K_cat_* was calculated with rGTP as substrate and poly C RNA as template (Table 1). The fastest enzyme was GII.7-RdRp which had an incorporation rate (*K_cat_*) of 0.238 s^−1^, followed by the four GII.4 RdRps; 2006b-RdRp, 2006a-RdRp, rGII.4-RdRp and GII.4 US95_96-RdRp, with incorporation rates of 0.209 s^−1^±0.054, 0.183 s^−1^±0.024, 0.168 s^−1^±0.024, 0.158 s^−1^±0.039, respectively. The slowest NoV enzyme was rGII.b-RdRp with an incorporation rate of 0.155 s^−1^±0.093 (Table 1). The two HCV RdRps, HCV 3a-RdRp and HCV 1b-RdRp, had a much lower incorporation rate compared to NoV RdRps with a *K_cat_* value of 0.003 s^−1^ for both (0.003 s^−1^±8.06×10^−5^ and ±1.06×10^−5^ for HCV 3a-RdRp and HCV 1b-RdRp, respectively).

#### Increased incorporation rate associated with a Lys291Thr mutation in the NoV RdRp

The first reported GII.4 associated pandemic occurred in 1995/1996. It was not until seven years later, in 2002, that a second GII.4 associated pandemic occurred. Since the 2002 pandemic, however, three more GII.4 associated pandemics have arisen (2004, 2006 and 2007). Interestingly, an increase in incorporation rate was observed in post 2002 pandemic RdRps (2006a-RdRp and 2006b-RdRp) compared to pre 2002 GII.4 RdRps (US95_96-RdRp [1995], rGII.4-RdRp [2001]) (p-value 0.022, Table 1). However, comparison of the mutation rates showed no differences between pre and post 2002 GII.4 RdRps.

**Figure 1:**
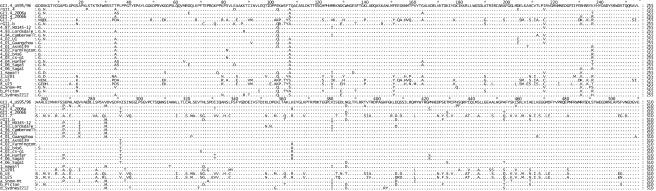
Comparison of the amino acid sequence of the six NoV RdRps used in this study to other representative GII strains. The top six sequences are the RdRps characterised in this study (GII.4 US95_96, rGII.4, GII.4 2006a, GII.4 2006b, GII.7 and rGII.b). The remaining 19 strains are reference strains and are named according to their GII genotype followed by their year of isolation (GII.4 strains only) and then their strain name. The alignment illustrated that a K291T substitution first appeared in the GII.4 lineage after 2001 and was unique for the GII.4 pandemic strains.

To identify specific residues associated with RdRps that had a higher incorporation rate, an alignment of the amino acid sequence of the six NoV RdRps was performed (Fig. 1). The three NoV RdRps with slower incorporation rates (GII.4 US95_96-RdRp, rGII.4-RdRp and rGII.b-RdRp) had a Lys at residue 291 whereas the three RdRps with faster incorporation rates had either a Thr (2006a-RdRp and 2006b-RdRp) or a Val (GII.7-RdRp) (Fig. 1) (mutation numbered according to Lordsdale virus RdRp, GenBank accession number X86557). No other amino acid variation was unique to the three faster enzymes (Fig. 1). A database search of GenBank for all full length NoV GII RdRp sequences available (accessed 9^th^ Nov, 2009) revealed the Thr291 residue was only identified in GII.4 strains isolated during or after 2001. In fact, the Lys291 mutation appears to have become fixed in the GII.4 pandemic lineage after its initial appearance in 2001.

**Figure 2:**
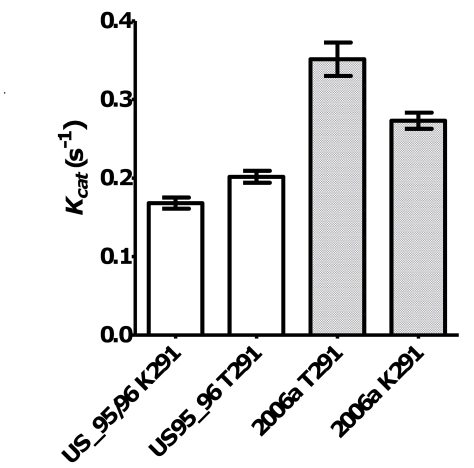
The effect of mutations at residue 291 on NoV GII.4 RdRp kinetic activity. A K291T mutation in wildtype (W+) NoV/US95_96-RdRp lead to a 20.2% increase in RdRp activity. Open bars show US95_96^291K^-RdRp: 0.168 s^−1^±0.018 [n = 6], US95_96^291T^-RdRp: 0.202 s^−1^±0.019 [n = 6)], p-value = 0.008. Whereas, a mutation T291K lead to a 22.2% reduction in activity for NoV/2006a-RdRp compared to the wildtype K291T. Hashed bars show 2006a^291T^-RdRp: 0.351 s^−1^±0.037 [n = 3], 2006a^291K^-RdRp: 0.273 s^−1^±0.020 [n = 4], p-value = 0.029.

To analyse the affect of the Lys291Thr mutation on the incorporation rate of the GII.4 RdRps, two mutant enzymes were made: GII.4 US95_96^K291T^-RdRp and GII.4 2006a^T291K^-RdRp. Specifically, the US95_96-RdRp 291Lys residue was mutated to a Thr and the 2006a-RdRp 291Thr was mutated to a Lys. The kinetic activity of the two mutant RdRps was then compared to the wildtype enzymes (Fig. 2). The Lys291Thr mutation increased US95_96-RdRp activity by 20.2% (US95_96^291K^-RdRp: 0.168 s^−1^±0.018 [n = 6], US95_96^291T^-RdRp: 0.202 s^−1^±0.019 [n = 6)] p-value = 0.008), whereas, the reverse Thr291Lys mutation decreased activity of the 2006a-RdRp by 22.2% (2006a^291T^ -RdRp: 0.351 s^−1^±0.037 [n = 3], 2006a^291K^ -RdRp: 0.273 s^−1^±0.020 [n = 4], p-value = 0.029) (Fig. 2).

#### In vitro analysis of the RdRp mutation rate

A high mutation rate (10^−3^ to 10^−5^ misincorporations per site) has been reported for most viral RdRps using either cell culture studies or biochemical analysis (reviewed in [20]). The mutation rate of the NoV RdRp has however, not been studied. Consequently, the present study developed an *in vitro* fidelity assay to enable direct comparison of the mutation rate of RdRps which could easily be applied for use with all non-culturable and culturable RNA viruses. Using this assay the mutation rate (substitutions per nucleotide site) for a transversion event (incorporation of UTP into a poly C RNA template) was calculated for all six NoV RdRps and for two control HCV RdRps (Table 1). The two HCV RdRps had the highest mutation rates of all eight enzymes at 1.60×10^−3^ (±0.52×10^−3^) substitutions per nucleotide site (Table 1). The mutation rates of the NoV RdRps were approximately one to two orders of magnitude lower than HCV (Table 1). The four GII.4 RdRps, US95_96-RdRp, rGII.4-RdRp, 2006a-RdRp and 2006b-RdRp, had similar mutation rates at 8.87×10^−4^, 8.34×10^−4^, 5.54×10^−4^ and 9.06×10^−4^ substitutions per nucleotide site, respectively, and were higher than the remaining two NoV RdRps, rGII.b-RdRp (1.53×10^−4^ substitutions per nucleotide site) and GII.7-RdRp (2.21×10^−5^ substitutions per nucleotide site) (Table 1).

The *in vitro* fidelity assay above examined transversion events, which are reported to occur at a lower frequency than transition events [20]. To confirm that the GII.4 enzymes have a higher mutation rate using different substrates the transition mutation rate was examined for two enzymes, US95_96-RdRp and GII.7-RdRp. Accordingly, the frequency of transition events (ATP into a poly C RNA template) was 1.5 and 1.7 fold higher than UTP (Table 1), for US95_96-RdRp, 1.30×10^−3^±1.08×10^−3^ (n = 3) and GII.7-RdRp, 3.71×10^−5^±1.21×10^−5^ (n = 3), respectively. This increase was not found to be significantly different from the transversion mutation rate (p-value = 0.5).

The *in vitro* transversion mutation rates were used to estimate the number of substitutions per viral genome replication event (U) for each RdRp [33] (Table 1). U equals the RdRp error rate multiplied by the genome size (7555 bp for GII.4 and GII.7, 7579 bp for GII.3 NoV and 9425 bp and 9408 bp for HCV 3a and 1b, respectively). The two HCV RdRps had the highest U values with an average of 15.07±4.96 substitutions per genome replication event (Table 1). The NoV RdRps had lower U values than HCV, with an average of 5.97±1.96 substitutions per genome replication event for the four GII.4 RdRps, 1.15 substitutions per genome replication event for rGII.b-RdRp and 0.17 substitutions per genome replication event for GII.7-RdRp (Table 1).

### Bioinformatic analysis of NoV capsid evolution

#### Strain selection

**Figure 3:**
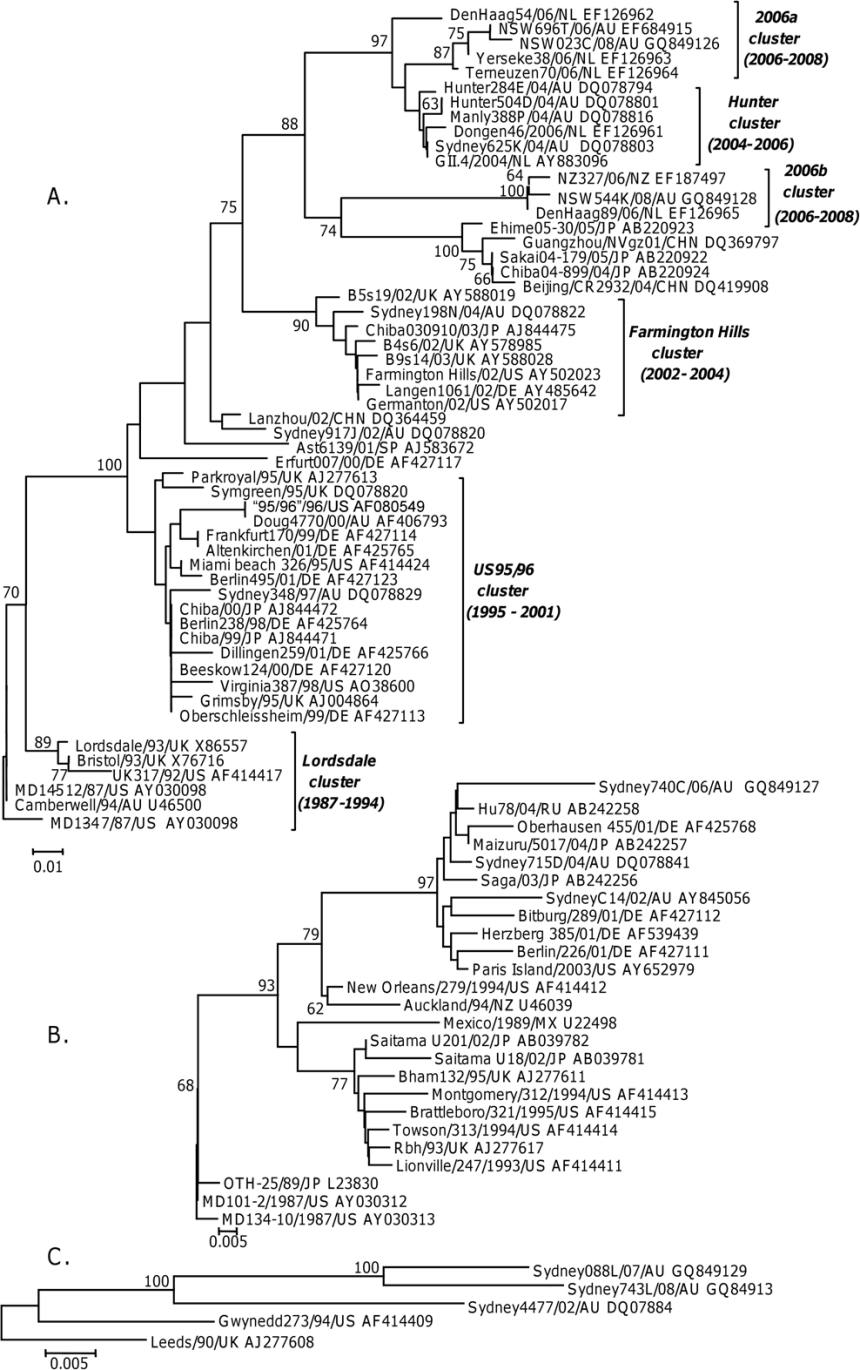
Phylogenetic analysis of the amino acid sequence of the P2 domain from GII.4 (A), GII.b/GII.3 and GII.3 (B) and GII.7 (C) strains circulating between 1987 and 2008. The phylogenetic tree was generated using the Neighbour-Joining method [51] by comparison of the P2 amino acid sequence (152 aa for GII.4 and 7, and 160 aa for GII.3) obtained for 84 NoV strains. GenBank accession numbers are included in the figure and follow the strain name. The percentage bootstrap values in which the major groupings were observed among 1000 replicates are indicated. The branch lengths are proportional to the evolutionary distance between sequences and the distance scale, in nucleotide substitutions per position, is shown.

The *in vitro* fidelity assay described above provides a format to directly compare the mutation rate of viral RdRps. To achieve a second independent comparison of the mutation rate for selected NoV GII genotypes, sequence data from four lineages was gathered and substitution rates were calculated by analysing sequence variation within ORF2, the capsid gene, over time. The capsid gene was chosen for two reasons; firstly, this region has the most sequence data available in nucleotide databases and secondly, ORF2 encodes VP1 which contains the host receptor binding domains that determine antigenicity of the virus and therefore provide the best indication of host driven evolution [14]. The four lineages examined were GII.4, GII.7, GII.3 and GII.b/GII.3 (Fig. 3A to C). The capsid lineage derived from the recombinant strain GII.b/GII.3 were analysed independently of the wildtype GII.3 lineage in order to examine the influence of the RdRp (ORF1) on rate of evolution of VP1.

The GII.4 capsid analysis included 54 GII.4 strains circulating between 1987 and 2008, with the oldest NoV strain MD134-7/87/US defined as the root (Fig. 3A). The GII.3 capsid analysis included 11 GII.b/GII.3 and 14 GII.3 strains circulating between 1987 and 2006, with MD134-10/87/US defined as the ancestral strain (Fig. 3B). Phylogenetic analysis indicated that the GII.b/GII.3 recombination event occurred prior to 2001 and the new recombinant virus subsequently evolved away from the wildtype GII.3 strains (Fig. 3B). Only five GII.7 strains with full length capsid sequence, three of which were generated in this study, were available for analysis. The five GII.7 strains were isolated between 1990 and 2007 and Leeds/90/UK was defined as the ancestral strain in this study (Fig. 3C).

#### Rate of evolution

**Figure 4:**
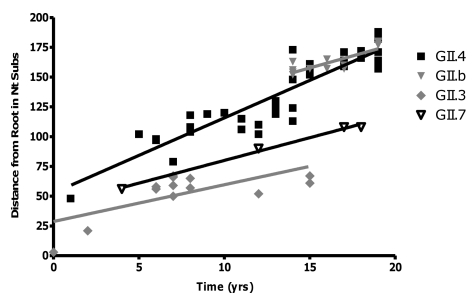
Rate of evolution for the GII.4, GII.7, GII.b/GII.3 and GII.3 strains. The rate of evolution for each genotype was determined by calculating the number of nucleotide substitutions in ORF2 compared to the oldest strain in each lineage. The number of changes was then plotted against the year of that strains detection. The rate of evolution was equivalent to the gradient of the line (GII.4 = 6.30±0.39, r^2^ = 0.84; GII.b = 4.03±0.80, r^2^ = 0.68; GII.3 = 3.09±0.95, r^2^ = 0.49; GII.7 = 3.82±0.25, r^2^ = 0.99) divided by the length of the capsid gene (1623 bp for GII.4 and GII.7, and 1647 bp for GII.3 and GII.b/GII.3).

Analysis of the sequence data revealed that GII.4 NoVs had the highest rate of evolution at 3.9×10^−3^ nucleotide substitutions/site/year (equivalent to 6.30±0.39 nucleotide changes/capsid/year) (r^2^ = 0.84, n = 54, p<0.0001) (Fig. 4). The rates of evolution for the wildtype GII.3 strain, the GII.b/GII.3 recombinant strain and the GII.7 strain were lower at 1.9×10^−3^, 2.4×10^−3^ and 2.3×10^−3^ nucleotide substitutions/site/year, respectively (r^2^ = 0.28, n = 14, p = 0.004; r^2^ = 0.63, n = 11, p<0.001 and r^2^ = 0.99, n = 5, p = 0.002) (Fig. 4). Statistical analysis of the data suggested that the GII.4 rate of evolution was significantly higher (p<0.010) than the GII.b/GII.3, GII.3 and GII.7 rates of evolution.

The amount of selection (purifying and positive selection) occurring in the capsid gene from the GII.4, GII.b/GII.3 and GII.7 strains was examined by calculating the Ka/Ks ratio for each genotype individually. The Ka/Ks ratio generated for the GII.4 strains was higher (0.0912±0.0322) than that of the GII.b/GII.3 strains (0.0862±0.495) and the GII.7 strains (0.0437±0.0235).

#### Evolution hotspots within the NoV capsid

**Figure 5:**
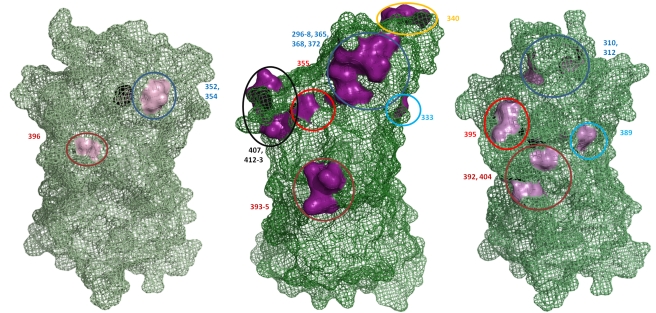
Hypervariable residues in GII.3, GII.4 and GII.7 are localised to common regions on the surface of the capsid P2 domain. The structure of the GII.4 P1 and P2 domain was solved previously (PDB ID 2OBS [52]) while the structure of the P domain was predicted for GII.3 and GII.7 in this study. The location of the hypervariable residues are indicated numerically and are coloured pink for all three genotypes. Residues occupying similar regions are depicted by the same coloured circle. The previously published hypervariable residues in the P2 domain of GII.4 were localised to six main regions on the surface of the P2 domain [4],[15]. GII.3 had hypervariable residues in four of these regions and GII.7 had hypervariable residues in two of these regions.

Sequence alignments of the capsid P2 domain from 54 GII.4 strains supported previously published data that there are 15 major amino acid residues which vary between each GII.4 pandemic cluster. These amino acids include 296 to 298, 333, 340, 355, 365, 368, 372, 393 to 395, 407, 412 and 413 (supplementary Fig. S1A) [4],[15]. Examination of the position of these 15 residues on the predicted secondary structure revealed that they clustered on the surface of six exposed loops of the P2 domain (Fig. 5). Similar amino acid alignments for GII.7 and GII.3 revealed there were three and six hypervariable sites which clustered onto two and four exposed loops of the P2 domain, respectively (supplementary Fig. S1B and C). A structural alignment revealed that the hypervariable residues in the GII.3 and GII.7 occupied overlapping spatial sites compared to the hypervariable residues in GII.4 described above (Fig. 5). In particular, the site occupied by 296 to 298, 365, 368, 372 in GII.4 corresponded to 310 and 312 in GII.3, 333 in GII.4 corresponded to 389 in GII.3, 393 to 395 in GII.4 corresponded to 392 and 404 in GII.3 and 355 in GII.4 corresponded to 395 in GII.3 (Fig. 5). GII.7 only had two variable regions, 352/354 and 396, and these corresponded to similar spatial orientation as the GII.4 variable sites 296 to 298, 365, 368, 372, and 393 to 395, respectively (Fig. 5).

## Discussion

Over the last decade five NoV pandemics have occurred approximately every two years and all pandemics have been associated with a single NoV genotype, GII.4 [16],[17],[19],[34]. The reason for the predominance of the GII.4 strains has been the subject of much speculation but is currently unknown primarily due to a limited understanding of NoV population dynamics and evolution [4],[15],[35].

Studies with other RNA viruses indicate that viral fitness is dependent on many factors, such as, viral mutation, replication efficiency, population size and host factors (reviewed in [2]). To date progress has been made in understanding the role host factors have on NoV prevalence with several studies indicating that variations in viral docking to the blood group antigens may affect infectivity of individuals within a population (reviewed in [36]). In particular, GII.4 viruses bind to all blood group antigens, whereas, GII.1 and GII.3 viruses bind fewer blood group antigens and this could account for higher prevalence of GII.4 viruses [36]. This paradigm however remains controversial, especially for GII NoV, as not all studies show an association between blood group antigens and clinical infection [37],[38],[39].

Apart from the host/viral interaction, no other factors have been affiliated with NoV fitness. Recent studies performed with poliovirus have shown that an increase in fidelity leads to less genetic diversity and subsequently a reduction in viral fitness and pathogenesis because of a reduced adaptive capacity of the virus [40],[41]. It has been hypothesised that viruses are fitter if they are able to produce a more robust (diverse) population (reviewed in [42],[43],[44]). In the current study we examined whether there was a link between epidemiological fitness, as defined by their incidence, and the rate and accuracy of viral replication.

In the present study error rates were assessed directly by examining the mutation rate of the viral RdRp and by analysing the rate of evolution for selected GII lineages. Our results are consistent with mutation rates for the poliovirus RdRp [26] and retrovirus reverse transcriptases [45], which range between 10^−3^ to 10^−5^ (Table 1). The more prevalent GII.4 strains had a 5 to 36-fold higher mutation rate compared to the less frequently detected GII.b/GII.3 and GII.7 strains, as determined by *in vitro* enzyme assays. Consistent with this, the rate of evolution of the capsid was on average 1.7-fold higher in GII.4 viruses compared to GII.3, GII.b/GII.3 and GII.7 viruses. The GII.4 capsids also had a larger Ka/Ks ratio than the GII.b/GII.3 and GII.7 strains suggesting that the increased incidence/epidemiological fitness of the GII.4 strains maybe through greater antigenic drift, a consequence of the higher mutation rate of the GII.4 RdRp.

The mutation rates for the control HCV RdRps (average of 1.6×10^−3^ substitutions per nucleotide site, Table 1) were 2-fold higher compared to the GII.4 RdRps. Evaluation of previously published rates of evolution for the HCV hypervariable region 1 (HVR1) within the envelope 2 glycoprotein (E2) were also higher (6–fold) than the NoV GII.4 rates of evolution calculated in this study [46] (Table 1). HVR1 was chosen for comparison because, like the NoV capsid gene, it is the most variable region in the genome and under the greatest immune selection. Mutation rate and rate of evolution cannot be directly compared as they are indirectly related due to the increased complexity of evolution *in vivo* [20]. However, in this study we did find a common trend between the two different measurements of diversity with HCV displaying the highest diversity rate for both measurements compared to NoV.

Interestingly, the majority of non-synonymous mutations in the P2 domain for all three NoV genotypes were localised to six common structural sites. These six hypervariable regions within the P2 domain were consistent with hypervariable sites for GII.4 capsids already identified in other studies [4],[19]. We demonstrated that GII.7 and GII.3 viruses shared two and four common hypervariable sites, respectively, with GII.4 viruses (Fig. 5). Substitutions at one of these sites (residue 395) have been shown to alter GII.4 strains antigenic profiles [4]. Localization of the hypervariable sites to common regions on the surface of the P2 domain suggests that these regions are likely to be under immune pressure possibly from a neutralizing antibody response [39]. The lower number of amino acid changes at these sites for viruses with a GII.3 capsid may explain why GII.b/GII.3 is predominantly associated with gastroenteritis cases in children [47]. This suggests that GII.b/GII.3 viruses are not as efficient at escaping herd immunity compared to GII.4 strains and therefore only hosts immunologically naïve to GII.3 infection are susceptible. Similarly, we propose that the low prevalence of the GII.7 strain is also a consequence of a low mutation rate in the RdRp resulting in limited antigenic drift and an inability to escape herd immunity.

Apart from mutation rate, replication rate is considered to be another major determinant in viral fitness [48]. Replication rates are important because an increased replication rate would produce a larger heterogenous population than a slower replicating virus in the same unit of time, given the same mutation rate. Interestingly, the RdRps from the recent 2006 GII.4 pandemic strains had a higher nucleotide incorporation rate than the recombinant GII.4 RdRp and the US95/96-like pandemic GII.4 RdRp, which could be associated with a point mutation in the RdRp (Thr291Lys). Residue 291 is located in the finger domain, which is comprised of five β sheets that run parallel and strongly interact with each other. The innermost of these five β sheets contains motif F which interacts directly with incoming nucleotides [49]. Therefore, it is plausible that substitutions at residue 291 affects the orientation of motif F due to the strong interaction between the five β sheets and subsequently alters the binding affinity to the incoming nucleotide. Fixation of the Thr291Lys point mutation in the GII.4 lineage after 2001 has been paralleled with a reduction in the period of stasis between the emergence of new antigenic variants [4]. Alterations in residue 291 after 2001 could have led to an increase in the rate of evolution of GII.4 strains by increasing the replication rate, however this did not seem to have an effect on mutation rate (Table 1). High replication rates did not always correlate with epidemiological fitness as the NoV strain, GII.7, had the highest incorporation rate but is considered to be the least fit due to it having the lowest incidence. Therefore, this study suggests mutation rate in combination with a high replication rate are key determinates in epidemiological fitness.

Influenza research also indicates a relationship between rate of evolution and epidemiological fitness (reviewed in [21]). New antigenic influenza A variants arise every one to two years and cause more annual epidemics than influenza B, as well as the more devastating pandemics [21]. Once a population has accumulated mass herd immunity to a virus the virus is forced to alter its antigenic determinants, a possibility for viruses with poor fidelity and fast replication rates, or face extinction [50], whereas, viruses such as influenza B, which have higher fidelity and slower antigenic change, are more often associated with sporadic cases [21]. In this study a parallel can be seen in the epidemiology between NoV and influenza, in particular between GII.b/GII.3 viruses and influenza B and GII.4 viruses and influenza A.

In summary, this study supports the hypothesis that epidemiological fitness is a consequence of the ability of the virus to generate genetic diversity, as the NoV pandemic GII.4 strains were associated with an increased replication and mutation rate. Therefore, it would seem that GII.4 viruses, as opposed to GII.b/GII.3 and GII.7 viruses, have reached a balance in their replication rate and mutation rate that is better suited to viral adaptation. In contrast, it would seem that the GII.7 lineage, despite having a high replication rate, has a low mutation rate that limits its adaptation and therefore its incidence. It is important to improve our understanding of the mechanisms underlying NoV epidemiological fitness as future pandemics are expected.

## Supporting Information

Text S1 GenBank accession numbers used in this study.
(0.04 MB PDF)

Figure S1 Alignment of the amino acid sequences of the P2 domain from A) GII.4 strains circulating between 1987 and 2006, B) GII.3 strains circulating between 1987 and 2006, and C) GII.7 strains circulating between 1990 and 2008. Sequences include the 152 aa of the P2 domain from 54 GII.4 strains and 5 GII.7 strains, and 160 aa of the P2 domain from 25 GII.3 or GII.b/GII.3 strains. Sequences were aligned using Mega 4.0. The NoV sequences included in the alignment are the same as in Fig. 4.
(6.93 MB TIF)
