# Correction: Rapid Evolution of Pandemic Noroviruses of the GII.4 Lineage

**DOI:** 10.1371/annotation/19042899-9f1b-4ccc-b13e-2a8faf19421b

**Published:** 2010-04-12

**Authors:** Rowena A. Bull, John-Sebastian Eden, William D. Rawlinson, Peter A. White

In the Author Contributions section, Peter A. White (PAW) should be listed as one of the persons who wrote the paper. 

