# Supplementary material for: Rapid Evolution of Pandemic Noroviruses of the GII.4 Lineage
Source: PLoS Pathog. 2010 Mar 26;6(3):e1000831. doi: 10.1371/journal.ppat.1000831 (PMC2847951; doi:10.1371/journal.ppat.1000831)
Supplement: Text S1 — GenBank accession numbers used in this study. (0.04 MB PDF) [file ppat.1000831.s001.pdf]

The GenBank (http://www.ncbi.nlm.nih.gov/Genbank/) accession numbers for the RdRp genes described in this paper are: AY237413 (NoV/Mc17/01/Th), DQ078829, (NoV/Sydney 348/97/AU), EF187497 (NoV/NZ327/06/NZ), EF684915 (NoV/NSW696T/06/AU), AY845056 (NoV/Sydney C14/02/AU), DQ078845 (NoV/Sydney4264/01/AU). The accession numbers for the capsid genes described in this paper are: EF126962 (Den Haag 54), EF684915 (NSW696T), GQ849126 (NSW023C), EF126963 (Yerseke38), EF126964 (Terneuzen70), DQ0787094 (Hunter284E), DQ078801 (Hunter504D), EF126961 (Dongen46), DQ078816 (Manly388P), DQ078803 (Sydney625K), AY883096 (GII.4/2004/NL), EF187497 (NZ327), GQ849128 (NSW544K), EF126965 (DenHaag89), AB220923 (Ehime05-30), DQ0369797 (Guangzhou/NVgz01), AB220922 (sakai04-179), AB220924 (Chiba04-899), DQ419908 (Beijing), AY588019 (b5s19), AJ844475 (Chiba030910), AY578985 (b4s6), AY588028 (b9s14), AY502023 (Farmington Hills), AY485642 (Langen1061), AY502017 (Germanton), DQ364459 (Lanzhou), DQ078820 (Sydney917J), AJ583672 (Ast6139), AF427117 (erfurt007), AJ277613 (Parkroyal), AJ277619 (Symgreen), AF080549 ("95/96"), AF406793 (Doug4770), AF427114 (Frankfurt170), AF425765 (Altenkirchen), AF414424 (Miami Beach 326), AF427123 (Berlin495), DQ078829 (Sydney348), AJ844472 (Chiba/00/JP), AF425764 (Berlin238), AJ844471 (Chiba/99/JP), AF425766 (Dillingen259), AF427120 (Beeskow24), A038600 (Virginia387), AJ004864 (Grimsby), AF427113 (Oberschleissheim), X86557 (Lordsdale), X76716 (Bristol), AF414417 (UK3), AY030098 (MD145-12), U46500 (Camberwell), AY030098 (MD134-7), AY030313 (MD134-10/1987/US), AY030312 (MD101-2/1987/US), L23830 (OTH-25/89/JP), AF414411 (Lionville/247/1993/US), AJ277617 (Rbh/93/UK), AF414414 (Towson/313/1994/US), AF414415 (Brattelboro/321/1995/US), AF414413 (Montgomery/312/1994/US), AJ277611 (Bham132/95/UK), AB039781 (Saitama U18/02/JP), AB039782 (Saitama U201/02/JP), U22498 (Mexico/1989/MX), U46039 (Auckland/94/NZ), AF414412 (New Orleans/279/1994/US), AY652979 (Paris Island/2003/US), AF427111 (Berlin/226/01/DE), AF539439 (Herzberg 385/01/DE), AF427112 (Bitburg/289/01/DE), AY845056 (SydneyC14/02/AU), AB242256 (Saga/03/JP), DQ078841 (Sydney715D/04/AU), AB242257 (Maizuru/5017/04/JP), AF425768 (Oberhausen 455/01/DE), AB242258 (Hu78/04/RU), GQ849127 (Sydney740C/06/AU), AJ277608 (Leeds), AF414409 (Gwynedd273), DQ078846 (Sydney4477), GQ849129 (Sydney088L) and GQ849130 (Sydney743L).
