# Supplementary figures and images for: Rapid Evolution of Pandemic Noroviruses of the GII.4 Lineage
Source: PLoS Pathog. 2010 Mar 26;6(3):e1000831. doi: 10.1371/journal.ppat.1000831 (PMC2847951; doi:10.1371/journal.ppat.1000831)

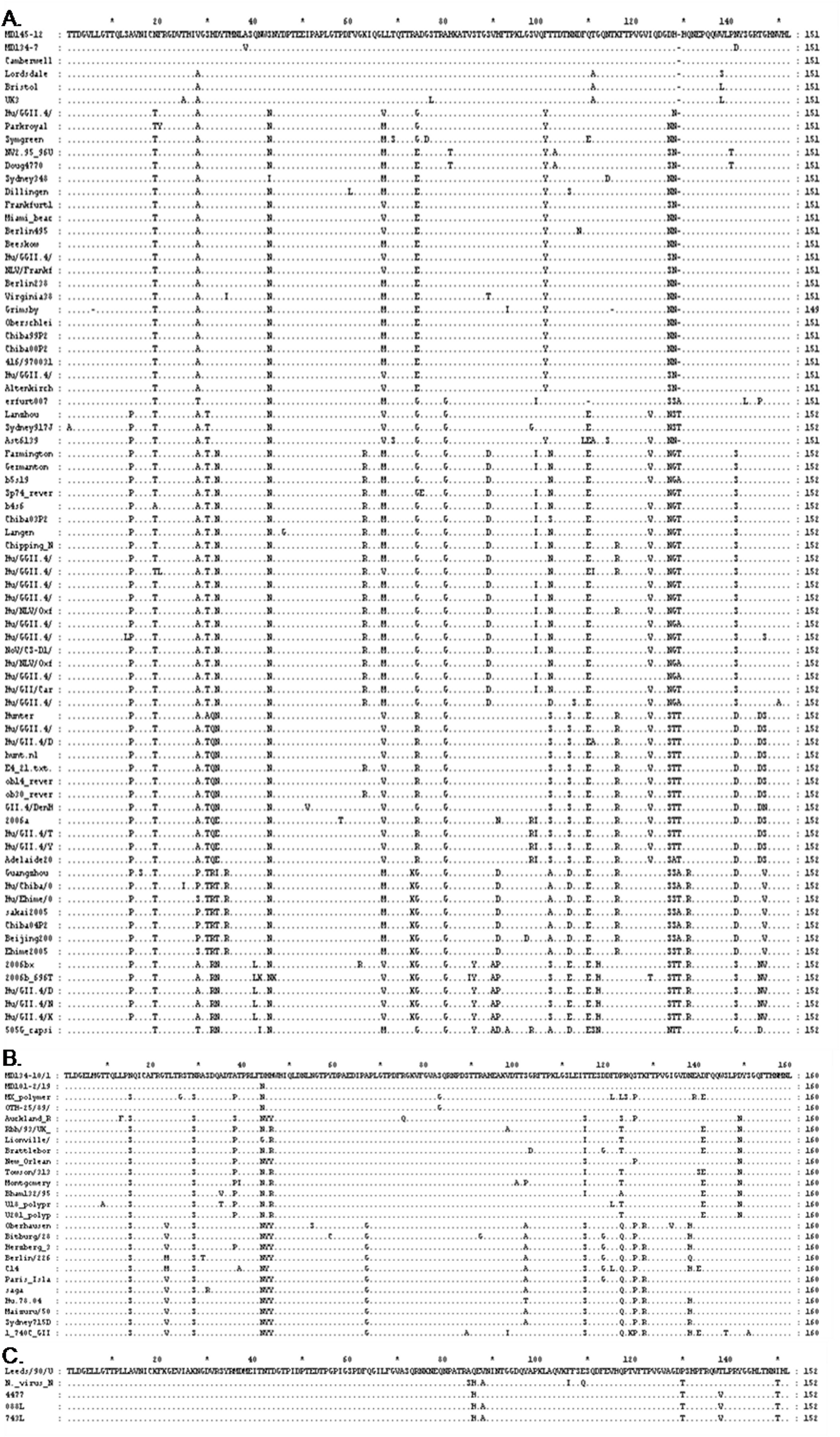

Supplement: Figure S1 — Alignment of the amino acid sequences of the P2 domain from A) GII.4 strains circulating between 1987 and 2006, B) GII.3 strains circulating between 1987 and 2006, and C) GII.7 strains circulating between 1990 and 2008. Sequences include the 152 aa of the P2 domain from 54 GII.4 strains and 5 GII.7 strains, and 160 aa of the P2 domain from 25 GII.3 or GII.b/GII.3 strains. Sequences were aligned using Mega 4.0. The NoV sequences included in the alignment are the same as in Fig. 4. (6.93 MB TIF) [file ppat.1000831.s002.tif]
